# Rethinking thresholds for serological evidence of influenza virus infection

**DOI:** 10.1111/irv.12452

**Published:** 2017-04-26

**Authors:** Xiahong Zhao, Karen Siegel, Mark I‐Cheng Chen, Alex R. Cook

**Affiliations:** ^1^ Saw Swee Hock School of Public HealthNational University of Singapore and National University Health SystemSingapore; ^2^ Communicable Disease CentreTan Tock Seng HospitalSingapore

**Keywords:** Bayesian modelling, diagnostic tests, influenza, longitudinal multinomial ordinal probit model, pandemic H1N1, serologic tests

## Abstract

**Introduction:**

For pathogens such as influenza that cause many subclinical cases, serologic data can be used to estimate attack rates and the severity of an epidemic in near real time. Current methods for analysing serologic data tend to rely on use of a simple threshold or comparison of titres between pre‐ and post‐epidemic, which may not accurately reflect actual infection rates.

**Methods:**

We propose a method for quantifying infection rates using paired sera and bivariate probit models to evaluate the accuracy of thresholds currently used for influenza epidemics with low and high existing herd immunity levels, and a subsequent non‐influenza period. Pre‐ and post‐epidemic sera were taken from a cohort of adults in Singapore (n=838). Bivariate probit models with latent titre levels were fit to the joint distribution of haemagglutination‐inhibition assay‐determined antibody titres using Markov chain Monte Carlo simulation.

**Results:**

Estimated attack rates were 15% (95% credible interval: 12%‐19%) for the first H1N1 pandemic wave. For a large outbreak due to a new strain, a threshold of 1:20 and a twofold rise (if pared sera is available) would result in a more accurate estimate of incidence.

**Conclusion:**

The approach presented here offers the basis for a reconsideration of methods used to assess diagnostic tests by both reconsidering the thresholds used and by analysing serological data with a novel statistical model.

## 1 Introduction

Estimates of infection rates are crucial to decision‐making and communication during an epidemic, for long‐term public health planning, and to assess past responses. Without an accurate gauge of the size and severity of an epidemic, it is challenging to prioritize interventions and services to mitigate impact.^1^, ^2^ In the 2009 H1N1 pandemic, limited data inflated predictions of severity in the early stages, leading in turn to what in hindsight was overreaction in many quarters.^3^ Lessons learned from the 2009 epidemic and its aftermath can be applied to influenza epidemics of strains both old and new.

Serological assessments can play a key role in assessing influenza outbreaks because they allow diagnosis of subclinical or misdiagnosed cases, and as a result, they provide the basis for estimates of an epidemic's impact, including, for example, estimates of hospitalization and mortality rates.^2^ Common assays, such as haemagglutination inhibition, typically bracket the antibody level to an interval between two dilutions, for instance positive at 1:20 but negative at 1:40. Two study designs are frequently used: cross‐sectional, in which a positive measurement beyond a specific threshold—which for influenza is usually set to 1:40 or 1:32—is taken to indicate recent infection,^4^ and longitudinal, in which a rise in the highest positive reading between successive serum collections of four or more times (often called a “fourfold rise,” although the interval censoring means the rise could actually be more modest) is typically assumed to reflect infection during that time interval. Cross‐sectional designs can utilize residual samples of serology collected for other purposes and are logistically much simpler to implement than longitudinal serum collections. They can allow, in principle, real‐time estimates of attack rates,^2^ but have documented weaknesses, such as potentially leading to negative estimates of attack rates,^5^ and while single cross sections may be accurate for a novel pathogen, repeated sampling is necessary if there is existing immunity in the population, or to track changes in incidence necessary for supporting real‐time estimates of severity.^2^

Traditionally, an HAI titre of 1:40 or more was chosen to indicate infection in cross‐sectional studies, and it was found to be associated with a reduction in attack rates that varies between 20% and 80% depending on age group and setting in influenza vaccination studies.^6^, ^7^, ^8^, ^9^ This choice is somewhat arbitrary. On the other hand, a fourfold rise is the currently used threshold in longitudinal studies and has been found to have sensitivity of ~80% relative to a basket of other diagnostics.^10^ Neither justification relates to overall diagnostic accuracy per se, however, which is the primary goal of analyses to determine attack rates, for surveillance or for intervention trials.

More robust analysis is made interesting by several complicating factors: (i) titres are interval‐censored, with intervals that are too broad to justifiably ignore; (ii) longitudinal studies require accounting for repeated measurements; (iii) titre distributions are typically too skewed to assume normality.

This study proposes a new statistical approach to estimate attack rates for paired sera. In this, multinomial ordinal probit models account for censoring and non‐normality by invoking a latent “titre propensity” and nonlinear threshold variables, while the titre propensity is made bivariate to account for within‐individual correlations in time. In addition, using this to estimate attack rates directly, we assess the sensitivity, specificity and overall accuracy of alternative versions of traditional thresholds, using three scenarios: (i) a new strain of influenza against which there is little pre‐existing immunity, (ii) an outbreak of seasonal influenza and (iii) a period with little influenza activity, using data from a community cohort established in equatorial Singapore.

## 2 Methods

### 2.1 Data

**Figure 1:**
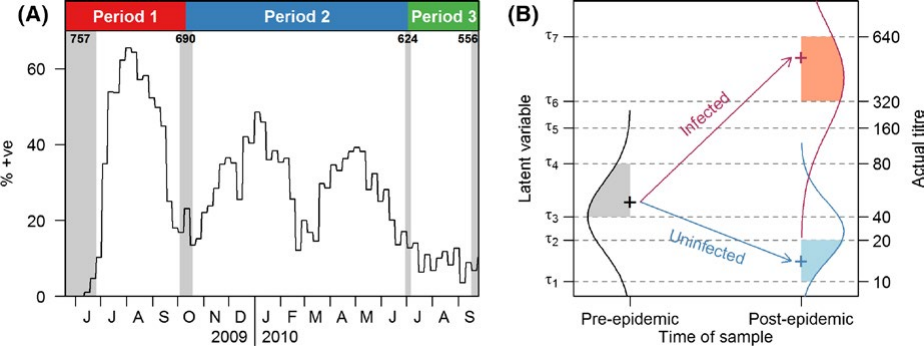
Schematic diagrams of data collection (A) and the developed longitudinal multinomial ordinal probit model (B). In panel A, the shaded grey areas indicate the period of serum sample collection, and the numbers indicate the number of samples collected. The black line represents the weekly relative proportions of influenza A(H1N1)pdm09 infections obtained from routine primary care surveillance. The top coloured panel indicates how we define the three periods, where period 1 refers to the first pandemic wave, period 2 refers to the two subsequent epidemic waves, and period 3 refers to the non‐influenza period. For panel B, in the pre‐epidemic phase, the continuous measure of antibody levels of each individual is represented by a latent variable which is bounded by the observed titre interval and is normally distributed (black normal curve). In the post‐epidemic phase, the latent variable for antibody levels of each individual still follows a normal distribution with the same variance, but there is a change in the normal mean that depends on the infection status (red curve for infected and blue curve for uninfected). Estimated threshold parameters, τ_*k*_, are the same for all time points

Repeated serological samples were drawn from a cohort of adults (aged 21‐75) participating in the Multi‐Ethnic Cohort (MEC) of the Singapore Consortium of Cohort Studies, a long‐term prospective cohort study, as described in detail elsewhere.^1^, ^11^, ^12^ Blood samples were collected at six different time points in 2009 and 2010, and this study uses sera collected at four of those points: (i) baseline samples collected before July 2009, thus predating unlinked community transmission of the pandemic, (ii) a sample around October 2009, after the first but before the second wave, (iii) a sample in July 2010, which followed the subsequent two epidemic waves of influenza A(H1N1)pdm09 and (iv) a sample in September 2010, 10‐12 weeks after the July 2010 sample. By the end of the study period, a total of 757, 690, 624 and 556 samples were obtained at the four time points, that is before July 2009, around October 2009, in July 2010 and in September 2010, respectively. The remaining two blood samples which were collected in the middle of period 1 and period 2 were not included in the analysis because they fell in the middle of outbreaks, and thus, it is hard to interpret attack rates involving them. A total of 38 subjects reported being vaccinated against influenza A(H1N1)pdm09 during the study period and were thus excluded from the analysis. This study focuses on three time windows: period 1 (spanning samples 1‐2) which bracketed the first wave and in which the population had low initial immunity levels; period 2 (samples 2‐3) which bracketed the second and third waves and in which the population had high initial immunity levels; and period 3 (samples 3‐4) which had little influenza activity (Figure 1A). A total of 758 participants who provided at least one blood sample at time points 1 and 2 (690 provided at both), 691 who provided at least one at time points 2 and 3 (544 provided at both) and 610 who provided at least one at time points at time points 3 and 4 (498 provided at both) were included in analysis. Participants gave informed consent, and the study was approved by the National University of Singapore Institutional Review Board.

All blood samples were titrated in twofold dilutions of phosphate‐buffered saline from 1:10 to 1:2560 and analysed to determine the antibody titre, which is the reciprocal of the highest dilution of serum where haemagglutination was inhibited.^11^ Titre values below the limit of detection were coded as <1:10, and a change in titre values from <1:10 to 1:10 was considered to be a twofold rise. Laboratory methods are detailed elsewhere.^11^

### 2.2 Statistical model

We developed a longitudinal multinomial ordinal probit model for HAI titres that incorporated latent variables for infection status and antibody propensity for each individual at both sample points (the slight modifications needed for those with a single observation are described later). A schematic diagram appears in Figure 1B. A latent variable, *z*_*it*_, represents a continuous measure of antibody levels of individual *i* at time point *t* and is modelled as being normally distributed. At time point 1, *z*_*i*1_~*N*($\mu_{i},\sigma^{2}$), where the mean $\mu_{i}$ varies between individuals, to account for correlations between time points, as $\mu_{i}$~*N*(*a*,*w*), and the variance $\sigma^{2}$ accounts specifically for observation error. At time point 2, *z*_*i*2_~*N*($\mu_{i}+\delta_{i},\sigma^{2}$), where the change in mean, δ_*i*_, is *N*(*b*+*c*, *ν*) if *i* was infected and *N*(*b*, *ν*) if not. Infection between the two time points happens with probability *p*, the primary estimand of interest.

The latent model is linked to the observations by an increasing sequence of threshold parameters, τ_*k*_, that control the mapping from latent space to titre space. Titre intervals are coded 1 for <1:10, 2 for 1:10 to 1:20, 3 for 1:20 to 1:40, etc., and the observed titre *T*_*it*_ for individual *i* at time point *t* is equal to *k* if τ_*k*‐1_≤*z*_*it*_<τ_*k*_ (Table 1). This model formulation provides the convenience of working with normally distributed variables while providing the flexibility to characterize the skewed and often bimodal distribution of HAI titres observed in many studies,^6^, ^13^ and although it requires estimating an additional parameter per observable HAI titre level, these can be modelled as constant in time. To ensure statistical identifiability, the two most extreme thresholds are set to τ_1_=0 and τ_8_=1, with intermediate threshold‐free parameters. Threshold parameters, τ_*k*_, are fixed to be same for the three time windows.

**Table 1:** Summary of the terminology of titres used in the study

*z*_*it*_	*Y*_*it*_	Censored titre	Conventional terminology
*z*_*it*_≤τ_1_	1	<1:10	<1:10
τ_1_≤*z*_*it*_<τ_2_	2	1:10 to 1:20	1:10
τ_2_≤*z*_*it*_<τ_3_	3	1:20 to 1:40	1:20
τ_3_≤*z*_*it*_<τ_4_	4	1:40 to 1:80	1:40
τ_4_≤*z*_*it*_<τ_5_	5	1:80 to 1:160	1:80
τ_5_≤*z*_*it*_<τ_6_	6	1:160 to 1:320	1:160
τ_6_≤*z*_*it*_<τ_7_	7	1:320 to 1:640	1:320
τ_7_≤*z*_*it*_<τ_8_	8	1:640 to 1:1280	1:640
τ_8_≤*z*_*it*_	9	>1:1280	1:1280

Conventional terminology typically refers to the maximum tested titre at which there is a positive reaction. This corresponds to a bracket that interval censors the “true” titre, if more dilutions were tested. *Y*_*it*_ is a coded version of the data; *z*_*it*_ is a latent variable that, together with the thresholds τ_*k*_, conceptually determines the censored titre observed.

Standard probability theory dictates that the joint distribution of the two latent variables is bivariate normal, conditional on infection status^14^:

$$\left(\begin{array}{c} z_{i1}\\ z_{i2} \end{array}\right)∼\left\{\begin{array}{cc} N\left(\left(\begin{array}{c} a\\ a+b+c \end{array}\right),\left(\begin{array}{cc} \left[w+\sigma^{2}\right] & w\\ w & \left[w+\nu +\sigma^{2}\right] \end{array}\right)\right) & \text{if}\;i\;\text{is infected or}\\ N\left(\left(\begin{array}{c} a\\ a+b \end{array}\right),\left(\begin{array}{cc} \left[w+\sigma^{2}\right] & w\\ w & \left[w+\nu +\sigma^{2}\right] \end{array}\right)\right) & \text{otherwise.} \end{array}\right.$$

This is then mapped to an observed titre *T*_*it*_=*k*, if τ_*k*‐1_≤*z*_*it*_<τ_*k*_ and *t*=1, 2. Note that the transformation from the latent variable's space to the observed titre allows the distribution to be distorted away from a normal distribution to reflect the empirical shape of the titre distribution. This is described in more detail in the Supporting Information.

As a consequence, the likelihood contribution from individual *i* given his or her infection status follows from the two‐dimensional cumulative distribution function of a bivariate normal distribution. Unconditional on infection status, the likelihood is instead a weighted average with weights *p* and 1−*p* for infected and uninfected distributions, respectively. For computational efficiency, we count the number of individuals with each combination of titres at the two time points and refer these to a multinomial distribution with probabilities determined by the foregoing description.

For individuals with observations at one time point only, the likelihood follows from the appropriate marginal distribution, either *z*_*i*1_~*N*(*a*, *w*+σ^2^) for time point 1 and a weighted average of *z*_*i*2_~*N*(*a*+*b*, *w*+*ν*+σ^2^) and *N*(*a*+*b*+*c*, *w*+*ν*+σ^2^) at time point 2. Again, we calculate these probabilities for each possible titre and refer them to a multinomial to obtain the likelihood function.

### 2.3 Sensitivity analysis

An important assumption in the above model is that the risk of infection is taken to be independent of baseline titres, which allows the infection probability parameter to represent average risk without knowing the titre distribution. An alternative formulation in which the probability is individualized to have a linear relationship to the titre category (on the logit scale), that is where logit(*p*_*i*_)=α+β*T*_*i*1_ and *T*_*i*1_, is the observed titre for individual *i* at time point 1, was also developed and used to assess the sensitivity of our findings to this assumption. The sensitivity model is much more computationally intensive because it requires the individual‐level serological data as the input.

### 2.4 Algorithm

All parameters were estimated via a Markov chain Monte Carlo routine, coded in R version 3.0.3 (R Foundation for Statistical Computing, Vienna, Austria)^15^ using 50 000 iterations with a thinning of five iterations. Uniform prior distributions for all parameters over their support were taken and subject to the constraint τ_*i*_<τ_*j*_ for *i*<*j*. We used a multinomial proposal distribution to update all parameters jointly. Convergence was assessed using trace plots of parameters and using the Geweke diagnostic.^16^ Point estimates are posterior means, and uncertainty intervals are 95% equal‐tailed credible intervals (CrI).

The posterior probability of infection for an individual with a specific post‐seasonal titre (for the first wave, i.e. in the absence of pre‐existing immunity), or for a specific risk in observed titre intervals (for either wave), was calculated directly from the fitted model using Bayes’ rule. For the former, *p*(*I*_*i*_|*T*_*i*2_)=*p*(*T*_*i*2_|*I*_*i*_)*p*(*I*_*i*_)/*p*(*T*_*i*2_), where *I*_*i*_ is the infection status for individual *i* and *T*_*i*2_ is the observed titre for individual *i* at time point 2, which can be derived from the marginal distribution of titres at time 2 in the presence or absence of infection. For the latter, a similar expression is used.

### 2.5 Diagnostic accuracy of existing thresholds

Sensitivity, specificity, positive and negative predictive values (PPVs and NPVs, respectively) and overall accuracy were calculated to assess the performance of diagnostic tests for various titre thresholds, the latter two based on the prevalence estimated from the MEC data. We derived sensitivity/specificity by calculating the probability of a positive/negative test in the presence/absence of infection. To assess accuracy of different thresholds in future, plausible epidemics, PPVs, NPVs and accuracies were calculated directly from the sensitivity and specificity as the hypothetical true prevalence was increased from 0 to 1. Sensitivity, specificity, PPV and NPV were all calculated from simulations from the developed model instead of using an objective measure, such as PCR‐confirmed infections. Bias between the modelled prevalence and the hypothetical true prevalence was also calculated to compare the accuracy of the currently used thresholds (1:40 or a fourfold rise) with the model we developed.

## 3 Results

### 3.1 Titre dynamics

**Figure 2:**
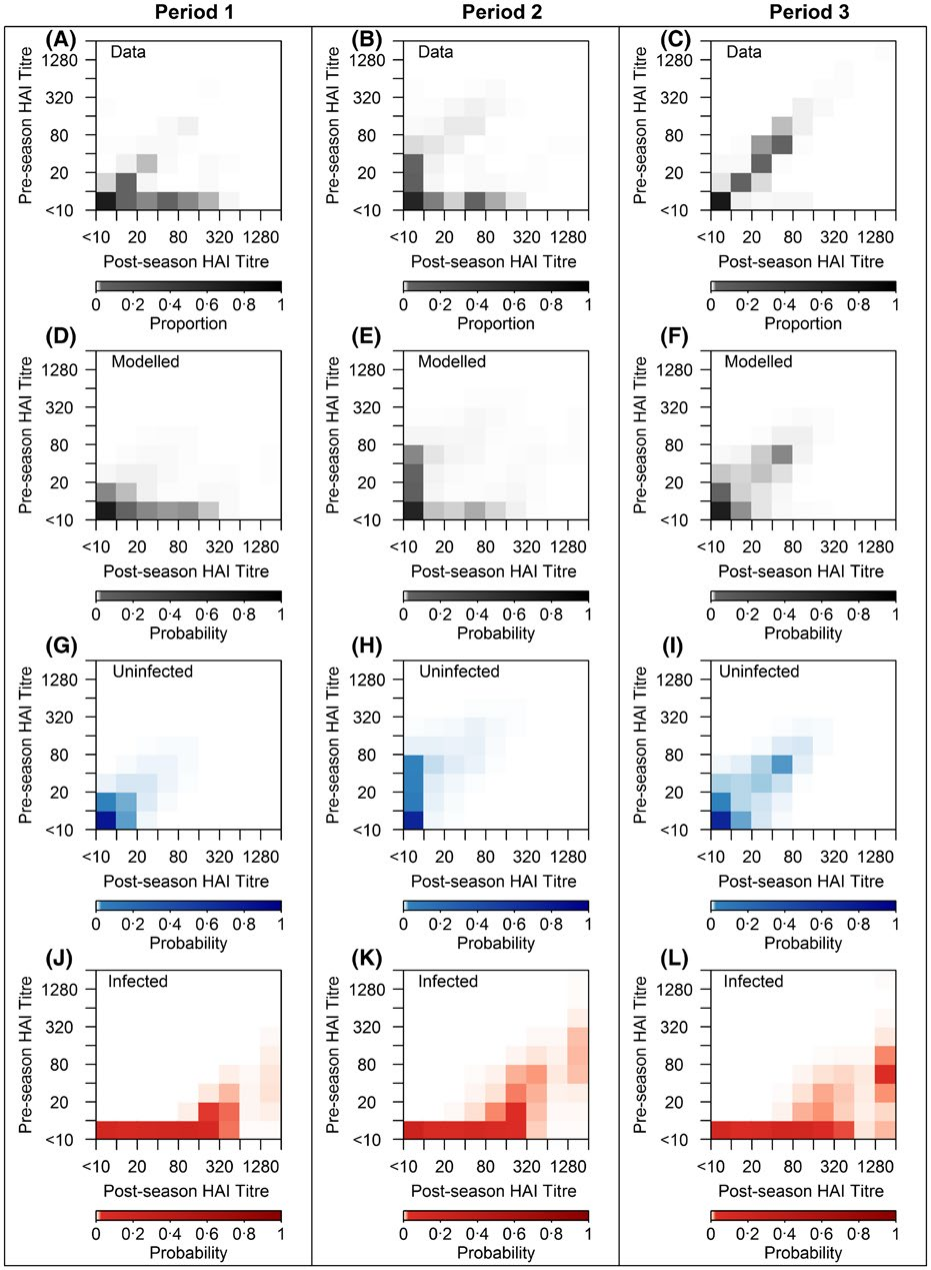
Distribution of observed haemagglutination‐inhibition (HAI) titres (A‐C), modelled titres (D‐F) and the proportion of uninfected (G‐I) and infected (J‐L) for HAI titres in periods 1‐3. A colour bar is placed at the bottom of each panel, with darker colour representing higher probabilities

Figure 2 illustrates the aggregate actual and modelled titre distributions as well as modelled titre distributions in the presence or absence of infection during the three time periods. The concordance between modelled and observed joint distributions was close for all periods. During the first wave (period 1), the majority of participants (79%) had no change in titre scores at the two time points, while the interval‐censored titres rose for 17% of participants and fell for the remaining 5% (Figure 2A). During the subsequent two epidemic waves (period 2), 62% participants had the same titre scores at the two time points, while 17% had higher and 21% had lower titre scores at the post‐season sampling (Figure 2B). During the non‐influenza period followed epidemic waves (period 3), 88% of participants had same titre scores at the two time points and 4% had higher and 8% had lower titre scores at the post‐season sampling (Figure 2C). In the absence of infection, 88%, 75% and 78% of participants had titre scores unchanged during the first pandemic wave, the subsequent two epidemic waves and the non‐influenza period, respectively (Figure 2G‐I). Most of the participants who were infected had pre‐season titres of less than 1:10 (86% for period 1, 73% for period 2, 74% for period 3) (Figure 2J‐L).

### 3.2 Attack rates over three periods

Our primary estimand is the attack rate from pre‐season to post‐season. This was estimated to be 15% (95% credible interval (CrI): 12%‐19%) during the pandemic first wave (period 1), 16% (95% CrI: 12%‐21%) in period 2 and 1.2% (95% CrI: 0.3%‐2.4%) during the non‐influenza period (period 3). In a sensitivity analysis, these results were broadly consistent with those using the more sophisticated model in which the risk of infection was related to initial titre levels (being 19% [95% CrI: 17%‐20%], 12% [95% CrI: 10%‐14%] and 0.6% [95% CrI: 0‐1.7%], respectively) with regard to the total number of infections across the three time periods, but with a slightly different distribution of when the infections occurred. Standard approaches, in contrast, gave estimates for the attack rate during the first wave (period 1) of 12% (95% CI: 9%‐14%) using a 1:40 threshold and of 12% (95% CI: 10%‐15%) using a fourfold rise, and of 10% (95% CI: 8%‐13%) and 2% (95% CI: 1%‐3%) for period 2 and 3, respectively, using fourfold rise as a proxy for infection.

### 3.3 Threshold positive predictive values and sensitivity

**Figure 3:**
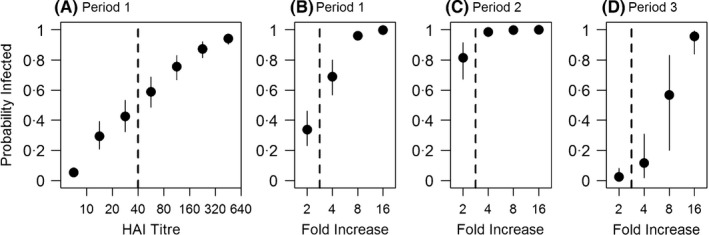
Infection probability by haemagglutination‐inhibition (HAI) titre in period 1 (A) and by fold increase in HAI titres in periods 1‐3 (B‐D). Points with whiskers, which indicate the 95% credible intervals, represent the posterior means

The estimated PPVs for cross‐sectional titres, that is the proportion of people with a titre of that level who were infected, were substantial even for low thresholds (Figure 3; Table S1): 0.43 (95% CrI: 0.31‐0.54) for a titre between 1:20 and 1:40, and 0.24 (95% CrI: 0.15‐0.34) for one between 1:10 and 1:20. In contrast, the PPV for a threshold of 1:40 and above was 0.81 (95% CrI: 0.73‐0.88). For a fourfold rise in titre scores, which is commonly used to reflect infection in longitudinal studies, the PPVs were 0.76 (95% CrI: 0.60‐0.88) and 0.92 (95% CrI: 0.77‐0.99) for periods 1 and 2, respectively, while it was only 0.03 (95% CrI: 0.01‐0.08) for the non‐influenza period (Figure 3; Table S2). Even for a rise of at least twofold in titre scores, the PPVs were high: 0.31 (95% CrI: 0.19‐0.45) and 0.68 (95% CrI: 0.43‐0.85) for the first and second periods, respectively, indicating that many infections are missed using the standard definition during the influenza epidemic waves. The difference in PPVs in different periods depends on the incidence of infection and the population herd immunity level, but the sensitivity estimates are quite consistent across the time periods, and thus epidemic scenarios, considered. The sensitivities of the traditional thresholds were as follows: period 1, 1:40: 0.60 (95% CrI: 0.49‐0.71); fourfold rise: 0.74 (95% CrI: 0.63‐0.83); period 2, fourfold rise: 0.64 (95% CrI: 0.55‐0.73); period 3, fourfold rise: 0.78 (95% CrI: 0.49‐0.96). The sensitivities for different HAI titre thresholds in different periods were summarized in Tables S3 and S4.

### 3.4 Threshold accuracy

**Figure 4:**
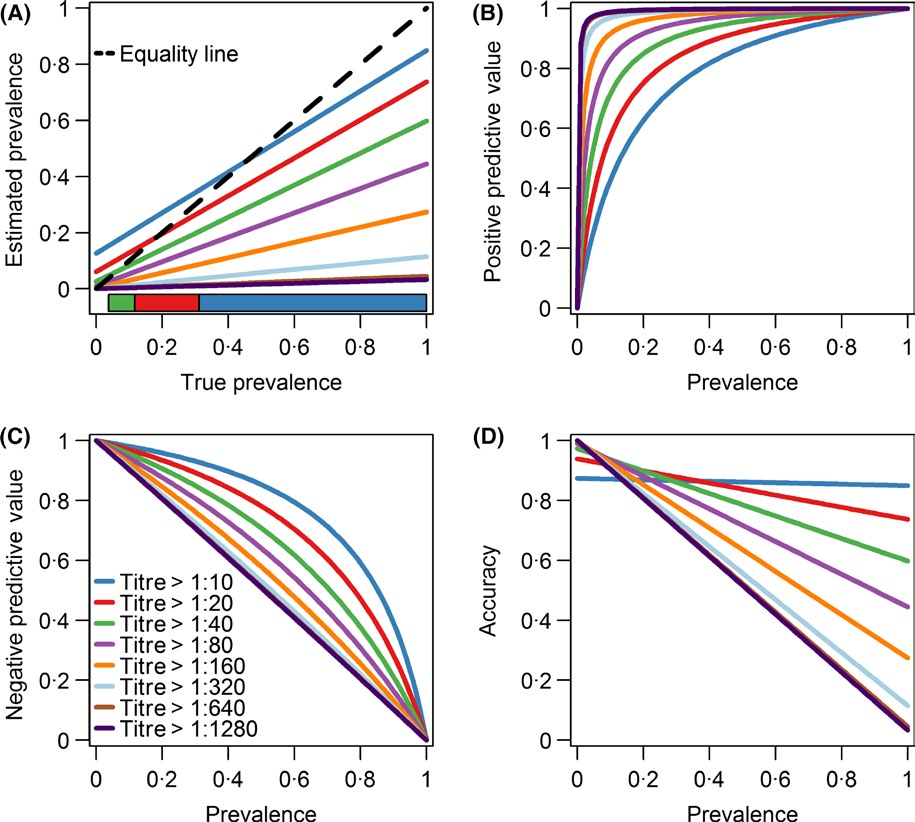
Estimated prevalence (A), positive predictive value (B), negative predictive value (C) and accuracy (D) by various titre thresholds in period 1. Different thresholds shown on each panel are plotted in different colours. On panel (A), the black 45‐degree equality dotted line describes an ideal scenario that the probability of a positive test equals to the probability of being infected. A colour bar is placed at the bottom of the panel (A) which shows the preferred titre threshold that should be used given the true prevalence. It is more preferred to use the threshold titre of 1:10 if the true prevalence is more than 0.31 as denoted by the dark blue polygon. The threshold titre of 1:20 should be used if the true prevalence falls between 0.12 and 0.32 as denoted by the red polygon. The threshold titre of 1:40 is preferred if the true prevalence falls between 0.04 and 0.12 as denoted by the green polygon

**Figure 5:**
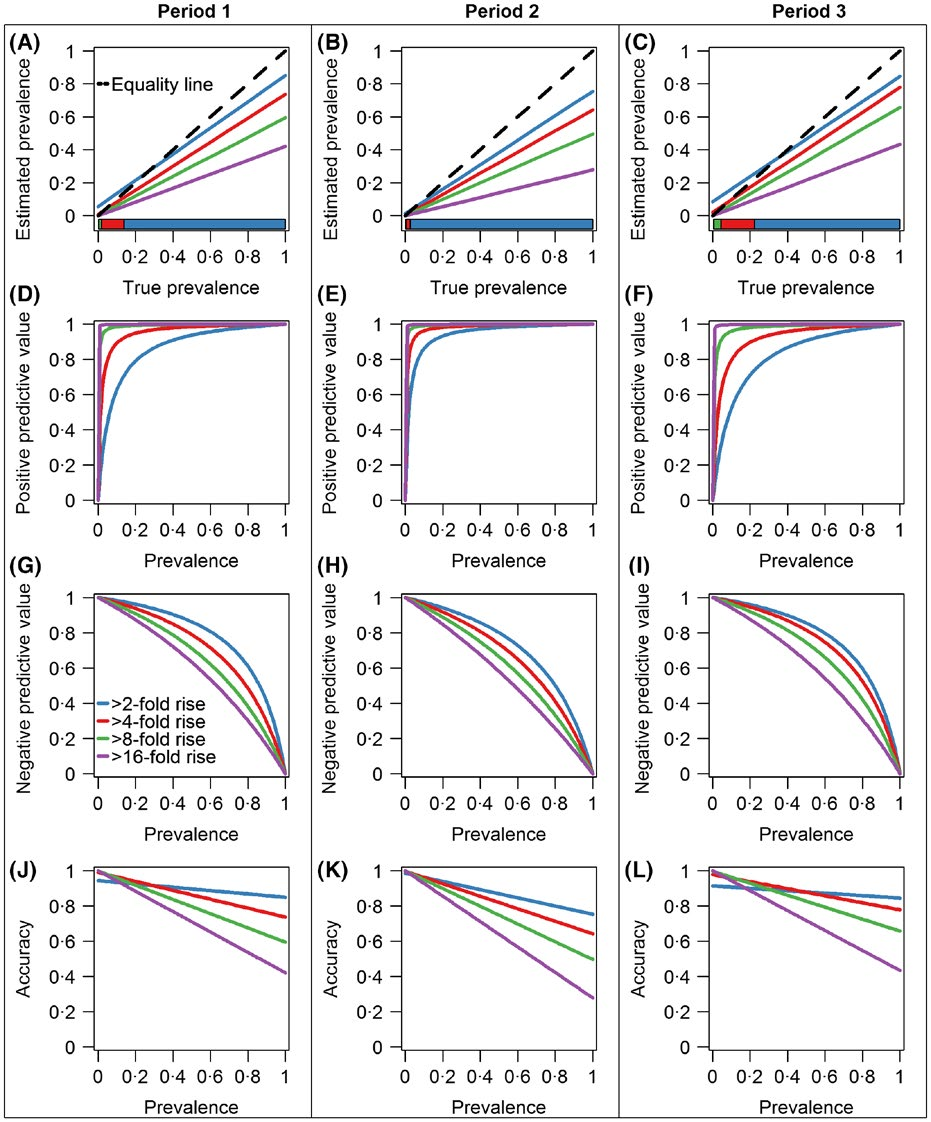
Estimated prevalence (A‐C), positive predictive value (D‐F), negative predictive value (G‐I) and accuracy (J‐L) by fold increase in titre scores in periods 1‐3. Different thresholds shown on each panel are plotted in different colours. On panel (A‐C), the black 45‐degree equality dotted line describes the ideal scenario that the probability of a positive test equals to the probability of being infected. A colour bar is placed at the bottom of the panel (A‐C) which shows the preferred fold increase threshold that should be used given the true prevalence

For straight‐forward prevalence estimates to be unbiased, the proportion of participants testing positive should equal the proportion infected, but for imperfect tests, this depends on the balance between sensitivity and specificity as well as the true prevalence. As depicted in Figure 4A, for cross‐sectional studies in an influenza pandemic first wave, based on our estimated sensitivity and specificity, the most accurate threshold would be the standard 1:40 only for small pandemics with an attack rate between 4% and 12%; for larger pandemics, this threshold leads to estimates that are biased downwards. This bias could be reduced using a lower threshold of 1:20 (for attack rates of 12%‐31%) or of 1:10 for a pandemic infecting a third or more of the population, as predicted by many simulation models^4^ and in line with the 1918 pandemic.^17^ Similarly, use of a fourfold rise as evidence of infection minimizes bias for small pandemics (of 2%‐14% attack rates) but underestimates the impact of larger pandemic first waves, while a twofold rise would be more accurate for pandemics infecting more than 14% of the study population (Figure 5A). These findings are similar when using the estimates from Singapore's subsequent two epidemic waves after the first pandemic wave (Figure 5B,C).

**Figure 6:**
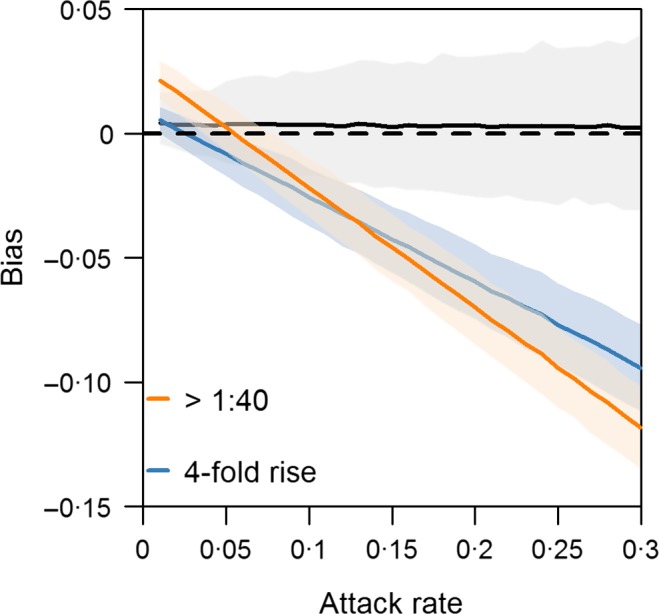
Degree of bias conditional on attack rate by threshold used for defining influenza infection. The black, orange and blue lines with shaded area, which indicates the 95% credible intervals, show the bias produced by the developed model, an haemagglutination‐inhibition (HAI) titre threshold of >1:40 and a fourfold rise in HAI titres, respectively

Figure 6 suggests the bias from standard thresholds in estimates of attack rates could be substantial. For a small new influenza pandemic infecting 15% of the population or a larger 1918‐like pandemic infecting 30% of the population, using the 1:40 threshold for a cross‐sectional study or a fourfold rise for a longitudinal study would substantially underestimate the numbers infected. On the other hand, using the model we developed would give much smaller bias regardless of overall prevalence variations in the community.

## 4 Discussion

Many studies using cross‐sectional serologic data for a newly circulating influenza strain use a haemagglutination‐inhibition (HAI) antibody titre of 1:40 or 1:32 as the standard threshold level, as studies have shown there to be ~50% decrease in the risk of infection with seasonal influenza viruses associated with a titre of 1:40.^18^, ^19^, ^20^ However, infected individuals may have HAI antibody titres of less than these levels.^5^ Similarly, many infected individuals do not experience the fourfold rise taken as evidence of seroconversion: the estimated sensitivity of this outcome versus a basket of other tests is ~80%.^10^ Our findings corroborates previous work by Cauchamez et al.^21^ who identified the importance of using a twofold rise for estimating attack rates, and that a fourfold rise might substantially underestimate attack rates. While either threshold is, depending on the study design, an unequivocal endpoint at the individual level for studies of risk of infection, for instance in vaccine trials^13^, ^22^ or risk factor studies,^23^ their imperfect sensitivity and specificity mean they may not adequately describe overall infection rates at a population level. As a result, estimates of severity may be similarly, and potentially substantially, biased.

We argue that there are three approaches to remedying this situation. One would be to use a statistical method that explicitly accounts for the structure of serological data—in particular, their censored nature, the distinct forms of statistical error (assay error and between individual variability) and the differential response over time of those infected and uninfected. The model presented in this study is an example of such, that can readily be fit using modern, if computationally intensive, statistical techniques. A second, simpler, approach would be to retain the use of a threshold but revise the level in response to the anticipated size of the outbreak. The results of the current analysis suggest that for a cross‐sectional study, the threshold should be set to 1:20 for outbreaks expected to infect >12% of the population and 1:10 for >32%, while for pre‐ and post‐outbreak sera, use of a twofold rise as a marker for infection would be more accurate for outbreaks >14%. Adapting this into a simple rule of thumb suggests that the existing thresholds should be retained for seasonal epidemics but lowered for all influenza outbreaks widespread enough to be classified as a pandemic. A third, simpler approach would be to use the traditional thresholds, that is ≥1:40 for a cross‐sectional study and ≥4‐fold rise for a longitudinal study, and adjust for the imperfect sensitivity of the assay. However, this approach would not be practical when the sensitivity is unknown without additional data.^1^

This recommendation warrants further investigation in other settings. However, taking our findings at face value suggests that substantially more people were infected during the 2009 pandemic than has been estimated^24^ and that severity metrics should be scaled up accordingly.

Previous research by Chen et al.^12^ using the same data set has shown that the new pandemic virus mostly affected the younger age group in Singapore (20‐29 years old). A stratified analysis by age group (<30 years old vs ≥30 years old) using the developed model has been carried out, and consistent results were obtained that younger age group showed higher attack rate during the first pandemic wave (19%; 95% credible interval: 11%‐30%) compared with the older age group (16%; 95% credible interval: 11%‐21%).

### Limitations

As the data analysed in this study were taken from a cohort study, it is not randomly selected and may not be representative of Singapore's population. This is a limitation common to most serological studies we have seen for influenza, with few exceptions (such as a large study in China^25^). The method we developed in this study depended upon the availability of serial serum sampling that begins prior to an outbreak, which can be costly and logistically complex, but which accounts for baseline antibodies present due to cross‐reactivity from different strains of a virus.^1^ The main analysis assumed the infection risk to be independent of baseline titre levels, albeit that this assumption is known to be untenable.^26^ Results from the sensitivity analysis that accounted for infection risk being influenced by initial titre were similar to those in the main analysis, but the alternative method was more computationally intensive. The developed model in this analysis only implicitly accounts for the waning effects in antibody titres over time, via a systematic reduction (or in principle increase) in mean latent titres between the time points. Cross‐reactivity is another potential limitation when analysing serologic tests to estimate the prevalence of seasonal influenza. For example, in the 2009 pandemic, there was a high level of pre‐existing seropositivity in older age groups due to cross‐reactivity, because the virus subtype had been endemic in the population prior to the 1957 influenza A(H2N2) pandemic.^2^ The approach further requires that the post‐pandemic sera are collected sufficiently soon after the end of epidemic activity such that titres have not decayed too substantially over time.^15^ There might be potential measurement errors in the titration of antibodies against A(H1N1)pdm09 infections; however, it cannot be assessed without duplicated assays at the same time point. Thresholds suggested from the current analysis in response to the anticipated size of the outbreak are specific to the context of the influenza A(H1N1)pdm09 outbreak, and further work is needed to demonstrate their validity for other epidemic scenarios.

Despite these limitations, these results challenge the predominant threshold of a 1:40 HAI titre or a fourfold rise in HAI titres and, in turn, the accuracy of many prior estimates of H1N1 attack rates. Precise estimates are important to public health planning and risk mitigation, and, therefore, a reconsideration of the standard paradigm should be considered.

## Conflict of interest

None declared.
