# Supplementary material for: Rethinking thresholds for serological evidence of influenza virus infection
Source: Influenza Other Respir Viruses. 2017 Apr 26;11(3):202–10. doi: 10.1111/irv.12452 (PMC5410725; doi:10.1111/irv.12452)
Supplement: Supplementary file 1 [file IRV-11-202-s001.docx]

**Supplementary material to Rethinking thresholds for serological infection in influenza**

**Authors:** Xiahong Zhao^1^, Karen Siegel^1^, Penny PX Goh^2^, Mark I-Cheng Chen^1,3^, Alex R. Cook^1,3,4^

**Affiliations:**

1 Saw Swee Hock School of Public Health, National University of Singapore and National University Health System, Singapore
2 Ministry of Education, Singapore
3 Communicable Disease Centre, Tan Tock Seng Hospital, Singapore
4 Program in Health Services and Systems Research, Duke-NUS Medical School, Singapore

**Author for correspondence:**
Alex R. Cook,
alex.richard.cook@gmail.com
Saw Swee Hock School of Public Health
Block MD1, National University of Singapore,
12 Science Drive 2, Singapore 117549

Text S1

For each study period, at least one blood sample was collected at the beginning or at the end of the period or both. All blood samples were assayed for hemaglutination-inhibition (HAI) titers against influenza infections. HAI titers are interval censored, either being negative at the lowest titration measured (1:10), positive at a titration of $1:X$ and negative at $1:2X$ (where $X$ falls in the set $\{10, 20, 40, 80, \ldots\}$, or positive at all titrations. In addition, the empirical titer distribution is far from a normal distribution, complicating analysis. To overcome this, a latent variable formulation was introduced, in which the latent variable was given a normal distribution and linked to observed HAI titers using a sequence of increasing threshold parameters. This formulation allows the modelling of HAI titer distributions using a normal distribution despite the actual measurements usually having a skewed or bimodal distribution. For notational simplicity, we relabel titer intervals as follows: <1:10 is relabeled 1, between 1:10 and 1:20 as 2, between 1:20 and 1:40 as 3 and so on.

At time point 1, i.e. at the beginning of period, the observed titer interval for individual $i$ was set to be $T_{i1}=k$ if $\tau_{k-1}\leq z_{i1}<\tau_{k}$, where $k\in\{1,2,\ldots\}$, and where the latent variable $z_{i1}$ was assumed to have a normal distribution, $z_{i1} \sim N\left( \mu_{i}, \sigma^{2} \right)$, with mean $\mu_{i} \sim N(a, w)$. The threshold parameters $\tau_{k}$ are also estimated and determine the mapping from latent to observed titer space. To ensure statistical identifiability, the threshold parameters, $\tau_{k}$, are set to be constant over time, and the two most extreme thresholds are set to $\tau_{1}=0$ and $\tau_{8}=1$. The latent variables are assumed to have a normal distribution with a mean, $\mu_{i}$, that accounts for the between-individual variation and is itself normally distributed with a mean $a$ and variance $w$, and $\sigma^{2}$ is the variance that accounts for random errors for each observation.

At time point 2, i.e. at the end of period, each individual would either become infected or stay uninfected. Infection between two time points is assumed to happen with probability $p$. The formulation for describing the distribution of HAI titers at time point 2 is an extension of the model for time point 1. The model mapping from the latent to observed space is taken to be the same, i.e. $T_{i2}=k$ if $\tau_{k-1}\leq z_{i2}<\tau_{k}$. The latent variable’s distribution is modified to be $z_{i2} \sim N\left( \mu_{i}+\delta_{i}, \sigma^{2} \right)$, where $\mu_{i}$ is the same as before, and $\delta_{i}\sim N(b+c,v)$ if $i$ was infected and $N\left( b,v \right)$ otherwise. The latter term, $\delta_{i}$, measures the change in mean for individual $i$ from time point 1 to time point 2, and it follows a normal distribution with variance $v$ and mean $b$ and $b+c$ for uninfected and infected respectively. Waning of titers is implicitly accounted for if $b$ is negative.

The final model and the likelihood contribution from an individual $i$ is, therefore, presented below in three different scenarios: (1) two blood samples collected both at the beginning and end of the period, (2) only one blood sample collected at the beginning of the period, and (3) only one blood sample collected at the end of the period. It is worth noting that individuals providing a single serum sample still contribute to the overall estimation, and do not therefore need to be excluded under this methodology, but we anticipate that a substantial fraction of individuals would need to provide two samples for the model to be able to provide reasonable estimates of attack rates.

**Two blood samples collected at the beginning and end of period**

The joint distribution of the two latent variables follows a bivariate normal distribution conditional on infection status. The covariance between two latent variables is $cov\left( z_{i1},z_{i2} \right)=cov(\mu_{i}+\epsilon_{i},\mu_{i}+\delta_{i}+\epsilon_{2})$ which is equal to $cov\left( \mu_{i},\mu_{i} \right)=w$ under the model’s assumption that $\mu_{i}, {\delta_{i}, \varepsilon}_{1}$ and $\varepsilon_{2}$ are independent of each other, where $\varepsilon_{1} \sim N(0,\sigma^{2})$ represents the random error for each observation at time point 1 and $\varepsilon_{2} \sim N(0,\sigma^{2})$ represents the random error at time point 2. With the derived covariance between two latent variables, we are able to determine the bivariate normal distribution for the two latent variables using the following equations: $T_{it}=k$ if $\tau_{k-1}\leq z_{it}<\tau_{k}$ for time point $t=1$ or 2, where

$$\binom{z_{i1}}{z_{i2}}\sim\left\{ \begin{matrix} N\left( \binom{a}{a+b+c},\left( {\left[ w+\sigma^{2} \right] \atop w}{w \atop\left[ w+v+\sigma^{2} \right]} \right) \right)\text{ if }i\text{ is infected or} \\ N\left( \binom{a}{a+b},\left( {\left[ w+\sigma^{2} \right] \atop w}{w \atop\left[ w+v+\sigma^{2} \right]} \right) \right)\text{ otherwise.} \end{matrix} \right.$$

The likelihood contribution from individual $i$ with two blood samples collected conditional on infection status follows from a two-dimensional cumulative distribution function of the above bivariate normal distribution. We use $\boldsymbol{l}_{12,i}$ and $\boldsymbol{l}_{12, u}$ to represent the likelihood contribution from individuals with or without infection respectively. Both $\boldsymbol{l}_{12,i}$ and $\boldsymbol{l}_{12, u}$ are a matrix of probabilities for each combination of titers at two time points. The likelihood unconditional on infection status is $\boldsymbol{l}_{12}=p\boldsymbol{l}_{12,i}+\left( 1-p \right)\boldsymbol{l}_{12, u}$, i.e. a weighted average. For computational efficiency, we count the number of individuals with each combination of titers at the two time points (represented by a 9 × 9 matrix $\boldsymbol{N}$) and refer these to a multinomial distribution with probabilities $\boldsymbol{l}_{12}$.

**Only one blood sample collected at the beginning of the period**

The likelihood, $\boldsymbol{l}_{1}$, for individuals with only one blood sample collected at time point 1 follows from a cumulative distribution function of the normal distribution, $z_{i1} \sim N\left( \mu_{i}, \sigma^{2} \right)$. We then count the number of individuals with each titer intervals at time point 1 and refer these to a multinomial distribution with probabilities $\boldsymbol{l}_{1}$.

**Only one blood sample collected at the end of the period**

The likelihood, $\boldsymbol{l}_{2}$, for individuals with observations at time point 2 only is a weighted average of the cumulative distributions of the two normal distributions with a probability of infection $p$: $z_{i2} \sim N\left( a+b, w+v+\sigma^{2} \right)$ for uninfected and $z_{i2} \sim N\left( a+b+c, w+v+\sigma^{2} \right)$ for infected. Again, we count the number of individuals with each titer intervals at time point 2 and refer these to a multinomial distribution with probabilities $\boldsymbol{l}_{2}$.

Note that because these are aggregate probabilities, the dataset can be collapsed into the total counts of each combination and referred to multinomial distributions, which simplifies the calculation of the likelihood function. In the variant model for the sensitivity analysis, the probability of infection becomes individual specific, namely

$$\log\frac{p_{i}}{1-p_{i}}=\alpha_{1}+\alpha_{2}T_{i1}.$$

This necessitates the calculation being carried out separately for each individual, or at least each starting titer, which we found increased computing time ~20-fold. Note also that for this model, we excluded those without a starting titer from the calculations because the distribution at the second time point could not be determined without more complex methods such as data augmentation.

All parameters were estimated via a MCMC routine and sampled from a multivariate normal proposal distribution. We adopted uniform prior distributions for all parameters over their support regions. In preliminary runs, a total of 10,000 iterations were performed with an arbitrary choice of variance-covariance matrix in the proposal distribution. Following this, the posterior means and a variance-covariance matrix for all parameters were estimated from sample, and then used as the mean and variance-covariance of a multivariate normal distribution as the proposal distribution for a subsequent 50,000 iterations with every 5^th^ iteration retained for final analysis.

Table S1. Positive predictive values by hemaglutination-inhibition titer interval during the first wave of influenza A(H1N1-2009) (period 1) in Singapore.

| Censored titer | Probability of infection (posterior mean (95% Credible interval)) |
| --- | --- |
|  | Period 1 |
| < 1:10 | 0.03 (0.01, 0.05) |
| 1:10 to 1:20 | 0.24 (0.15, 0.34) |
| 1:20 to 1:40 | 0.43 (0.31, 0.54) |
| 1:40 to 1:80 | 0.64 (0.53, 0.75) |
| 1:80 to 1:160 | 0.82 (0.72, 0.89) |
| 1:160 to 1:320 | 0.93 (0.87, 0.97) |
| 1:320 to 1:640 | 0.98 (0.95, 0.99) |
| 1:640 to 1:1280 | 0.99 (0.97, 1.00) |
| 1:1280 to 1:2560 | 1.00 (0.98, 1.00) |

Table S2. Positive predictive values by fold increase in hemaglutination-inhibition titer scores in period 1–3 in Singapore.

| Fold increase | Probability of infection (posterior mean (95% Credible interval)) | | |
| --- | --- | --- | --- |
|  | Period 1 | Period 2 | Period 3 |
| 2 | 0.31 (0.19, 0.45) | 0.68 (0.43, 0.85) | 0.01 (0.00, 0.01) |
| 4 | 0.76 (0.60, 0.88) | 0.92 (0.77, 0.99) | 0.03 (0.01, 0.08) |
| 8 | 0.98 (0.83,0.99) | 0.99 (0.95, 1.00) | 0.36 (0.12, 0.65) |
| 16 | 1.00 (1.00, 1.00) | 1.00 (0.99, 1.00) | 0.96 (0.87, 0.99) |

**Table S3. Sensitivity by hemaglutination-inhibition titer interval during the first wave of influenza A(H1N1-2009) (period 1) in Singapore.**

| Censored titer | Posterior mean sensitivity (95% Credible interval) |
| --- | --- |
|  | Period 1 |
| < 1:10 | 1 |
| 1:10 to 1:20 | 0.85 (0.77, 0.92) |
| 1:20 to 1:40 | 0.74 (0.63, 0.83) |
| 1:40 to 1:80 | 0.60 (0.49, 0.71) |
| 1:80 to 1:160 | 0.44 (0.35, 0.55) |
| 1:160 to 1:320 | 0.27 (0.20, 0.35) |
| 1:320 to 1:640 | 0.12 (0.07, 0.17) |
| 1:640 to 1:1280 | 0.05 (0.01, 0.09) |
| 1:1280 to 1:2560 | 0.03 (0.01, 0.08) |

Table S4. Sensitivity of infection by fold increase in hemaglutination-inhibition titers in period 1–3 in Singapore.

| Fold increase | Posterior mean sensitivity (95% Credible interval) | | |
| --- | --- | --- | --- |
|  | Period 1 | Period 2 | Period 3 |
| 2 | 0.84 (0.76, 0.92) | 0.75 (0.67, 0.83) | 0.84 (0.63, 0.98) |
| 4 | 0.74 (0.63, 0.83) | 0.64 (0.55, 0.73) | 0.78 (0.49, 0.96) |
| 8 | 0.59 (0.48, 0.71) | 0.50 (0.39, 0.60) | 0.66 (0.28, 0.92) |
| 16 | 0.42 (0.32, 0.53) | 0.28 (0.19, 0.38) | 0.43 (0.08, 0.82) |
